# A rare presentation of angiolymphoid hyperplasia with eosinophilia (ALHE) on the cheek: Case report and two-year follow-up

**DOI:** 10.1016/j.ijscr.2025.110953

**Published:** 2025-01-24

**Authors:** Tahmineh Tahouri, Ehsanollah Rahimi-Movaghar, Sahand Hedayati-Omami, Maryam Moeini

**Affiliations:** a Pediatric Cardiology, Cardiovascular Research Center, Rajaie Cardiovascular Institute, Tehran, Iran; b Department of Surgery, Farhikhtegan Hospital, Faculty of Medicine, Tehran Medical Science, Islamic Azad University, Tehran, Iran; c School of Medicine, Azad University, Tehran, Iran; d Clinical and Surgical Pathologist, Department of Pathology, Chamran Hospital, Tehran, Iran

**Keywords:** Angiolymphoid hyperplasia with eosinophilia, Eosinophilia, Vascular neoplasms, Surgical excision

## Abstract

**Introduction and importance:**

Angiolymphoid hyperplasia with eosinophilia (ALHE) is a rare benign vascular tumor characterized by abnormal endothelial proliferation and inflammatory cell infiltration, primarily affecting the head and neck region. The diverse clinical presentations of ALHE pose significant diagnostic challenges, often leading to misdiagnosis. Accurate histopathological examination is crucial for differentiating ALHE from other vascular lesions and guiding appropriate treatment.

**Case presentation:**

We report the case of a 27-year-old male who presented with a solitary, asymptomatic, dome-shaped lesion on his right cheek. Initial clinical examination and MRI suggested a diagnosis of atypical hemangioma. Surgical excision of the lesion was performed, and histopathological analysis revealed features consistent with ALHE, including vascular hyperplasia, epithelioid endothelial cells, and a mixed inflammatory infiltrate with eosinophils. The patient remained asymptomatic with no recurrence during a two-year follow-up period.

**Clinical discussion:**

This case highlights the diagnostic complexities associated with ALHE due to its varied clinical and radiological presentation, often mimicking other benign or malignant vascular lesions. Although MRI findings initially suggested hemangioma, histopathological confirmation was pivotal in establishing the correct diagnosis. The involvement of the cheek in ALHE is rare, with most cases affecting other regions of the head and neck. The standard treatment for ALHE remains surgical excision, with our patient showing no recurrence over a two-year period.

**Conclusion:**

Given the potential for misdiagnosis due to the overlapping features of ALHE with other vascular lesions, clinicians should maintain a high index of suspicion and consider histopathological examination in atypical cases.

## Highlights

• ALHE is a rare benign tumor with vascular proliferation and immune cell buildup.
• Common in the head and neck, ALHE may cause pruritus, pain, or bleeding.
• Cheek localization in ALHE is exceedingly rare, making this case notable
• Diagnosis confirmed via histopathology; MRI initially suggested hemangioma.
• Surgical excision is preferred for ALHE, with low recurrence when properly managed.

## 1 Introduction

Angiolymphoid hyperplasia with eosinophilia (ALHE) is a rare benign tumor featuring vascular proliferation and infiltration of lymphocytes and eosinophils [1,2]. Clinically, ALHE typically presents as smooth-surfaced, pink to violaceous papulonodules, most often localized to the head and neck, especially around the auricular area [1]. The incidence of ALHE is not well-documented but appears more frequent in Japan. Common symptoms include pruritus, pain, or bleeding [2,3]. Primary treatments include surgical excision or laser therapy, with a noted tendency for recurrence [4]. Here, we present the case of a 27-years-old male with a single asymptomatic ALHE lesion on the cheek. This case report is notable due to the rarity of ALHE and the diagnostic challenges it presents, contributing to a better understanding of the clinical manifestations and pathological features of this lesion.

This report has been prepared in accordance with the SCARE 2023 guidelines for reporting surgical case reports [5].

## 2 Case presentation

A 27-year-old male patient visited the surgical clinic with concerns about a progressively enlarging lesion on his right cheek, observed over the past two months. The lesion was painless, skin-colored, and had a smooth surface. His medical history was unremarkable, with no significant prodromal symptom, weight loss, appetite changes, or familial history of similar conditions. Additionally, there was no history of trauma to the affected area. The patient's primary reason for seeking medical attention was the increase in the lesion's size.

Clinical examination revealed a dome-shaped, nodular subcutaneous lesion on the right cheek, measuring approximately 2 × 2 cm. The lesion was firm, painless, non-tender, and without a palpable pulse. There was no difference in temperature between the lesion and surrounding skin. Examination of the head and neck lymph nodes yielded unremarkable results, and other clinical findings were within normal limits as well. Further investigations included complete and differential blood counts, kidney and liver function tests, urinalysis, serum electrolyte levels, and both contrast and non-contrast MRI of the face.

**Fig. 1:**
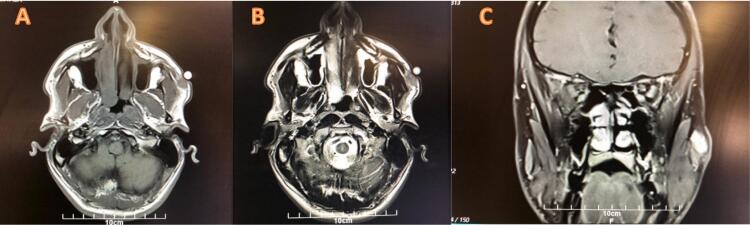
Pre-surgical MRI before surgery reveals a subcutaneous soft tissue lesion in the right zygomatic region, measuring 16*12*15 mm. The lesion exhibits isointense signal relative to muscle on T1- weighted imaging (T1W1) (A) and has intermediate to high signal intensity on T2-weighted imaging (T2W1) (B). The lesion shows vivid enhancement on the post-contrast MRI study (C).

Laboratory results revealed normal findings with no abnormalities detected. Hemoglobin levels and blood cell count were within expected ranges, and serum electrolyte levels, as well as liver and kidney functions showed no irregularities. However, MRI results indicated an ill-defined subcutaneous lesion in the right cheek, characterized by an isointense T1 signal and a hyper-intense T2 signal, with significant contrast enhancement, measuring 16 × 15 × 12 mm (Fig. 1). Based on these MRI findings, an atypical hemangioma was considered as a probable diagnosis. Consequently, a decision was made to proceed with excisional surgery. The patient underwent surgery one month after the initial consultation.

The surgery performed under general anesthesia and included preoperative prophylaxis with 1 g of cefazolin administered intravenously. The subcutaneous lesion was excised, and the specimen was sent for pathological examination. Macroscopically, a lesion measuring 2 × 2 × 2 cm was observed, comprising a piece of grayish subcutaneous tissue with a hard consistency, without involvement of the skin.

**Fig. 2:**
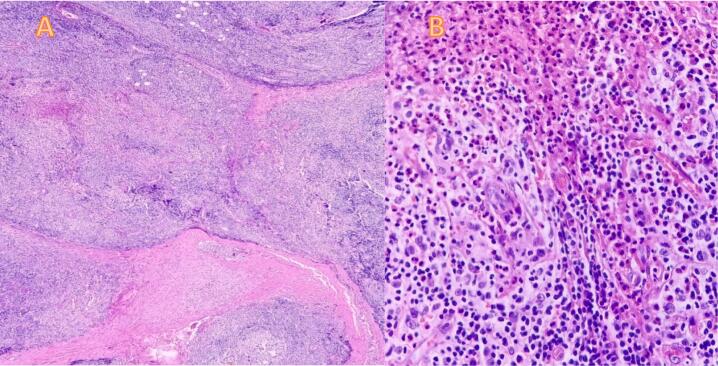
Histopathologic examination of the right cheek lesion reveals a proliferation of vascular channels accompanied by a mixed inflammatory infiltrate. The findings include nests and cords of endothelial cell proliferation, with an admixture of lymphocytes, plasma cells, and eosinophils (A). Microscopic view of eosinophils (B).

Microscopic analysis revealed vascular hyperplasia with epithelioid endothelial cells, along with an infiltrate of lymphocytes and eosinophils. Additionally, a few plasma cells and giant cells were observed (Fig. 2). Contrary to the MRI suggestion, the pathology report confirmed a diagnosis of angiolymphoid hyperplasia with eosinophilia.

The postoperative course was uneventful, and the patient was discharged one day after the surgery without the need for any prescribed medication. The patient was followed for 2 years, during which he remained asymptomatic, and no recurrence of the lesion was observed.

## 3 Discussion

Angiolymphoid hyperplasia with eosinophilia (ALHE), also referred to as epithelioid hemangioma (EH), is an inflamed vascular tumor of uncertain etiology. It is characterized by the proliferation of histiocytoid endothelial cells along with prominent lymphocytic and eosinophilic infiltration**.** Historically, it has been referred to by previous terms, including “pseudopyogenic granuloma”, “angiomatous nodule”, and “histiocytoid hemangioma” [6].

The precise etiology of ALHE remains uncertain, with several theories suggesting reactive, neoplastic, or infectious origins. Although debates persist regarding its classification as either a neoplastic process or an unusual hypersensitivity reaction, current evidence leans toward the latter. Certain reports propose that trauma or infections may also contribute to its development. The hallmark inflammatory infiltrate, a defining feature of ALHE/EH, is believed to play a role in its pathology, though its precise contribution is still unclear**.** It has been hypothesized that vascular proliferation arises from endothelial cells reacting to proliferative signals triggered by inflammatory cells or allergic responses. Additional factors like arteriovenous shunting, localized trauma, or elevated serum estrogen levels might also play a role [7]. Notably, our patient did not report any recent trauma to the affected region.

A mutation in the TEK gene, encoding the Tie-2 receptor on endothelial cells, has been identified in a case of dermal ALHE/EH. This suggests that molecular alterations could contribute to the condition's pathogenesis, although more comprehensive studies are required to confirm this link [7].

Geographically, ALHE is more frequently observed in Asian countries, particularly China, Japan, and South Korea [8]. While it is slightly more prevalent in females, studies within Asian populations often show a male predominance. Typically, ALHE occurs in individuals aged 20–50 years, with the average age of onset being 30–33 years. It is rarely reported in elderly individuals or non-Asian pediatric patients [7].

Clinically, ALHE typically presents as single or multiple papules or nodules, ranging in color from flesh to plum, and varying in size from a few millimeters to several centimeters [6,9]. The time from lesion appearance to seeking medical attention may span months to years. While these lesions are often asymptomatic, their vascular nature can result in tenderness, pulsation, itching, or bleeding, particularly after minor trauma. Peripheral eosinophilia and regional lymphadenopathy have also been reported in some cases [6]. To our knowledge, the largest systematic review of ALHE by Adler et al. [2], involving 908 cases, found a significant correlation between multiple lesions and symptoms such as pruritus and bleeding. In contrast, our patient was asymptomatic, with no lymphadenopathy and a normal eosinophil count.

Most papulo-nodular lesions associated with ALHE are found in the dermis and/or subcutis. Extracutaneous involvement has been documented in various sites, including the radial artery, colon, lacrimal gland, bone, and oral mucosa [1]. Approximately 53.4 % of ALHE lesions are solitary, while multiple lesions are often confluent of clustered [1,2].

While ALHE lesions most frequently appear on the head and neck—particularly the scalp and auricle, which account for approximately 87 % of cases [10]—our patient presented with a lesion on the cheek, an unusual site. A literature review identified only three previously documented cases of ALHE affecting the cheek: a 49-year-old male reported by Kown et al. [11], a 51-year-old male described by Moharraqi et al. [7], and a 21-year-old male detailed by Ingram et al. [12]. In the case reported by Al Moharraqi et al., the patient had a solitary mass measuring 8 × 6 cm on the right cheek, located subcutaneously above the upper lip and lateral to the nostril. Over three years, the lesion gradually increased in size but remained without ulceration, crusting, or discoloration. Physical examination revealed a firm, immobile, smooth lesion with no tenderness or regional lymphadenopathy. Kown et al. described a patient with a single nodule on the right cheek that appeared spontaneously two months before presentation. The lesion measured 1.5 × 1.5 cm and was soft and round, with unremarkable overlying skin and no pulsation. In the third case, reported by Ingram et al., the lesion was a 2 cm fibrous mass within the subcutaneous tissue of the cheek [7,11,12].

The diagnosis of ALHE is particularly challenging due to its place within an evolving, undefined spectrum of vascular tumors. Conditions that can present similarly include Kimura disease, epithelioid hemangioendothelioma, angiosarcoma, eosinophilic granulomatosis with polyangiitis, and juvenile temporal arteritis (JTA). Differential diagnoses for subcutaneous head lesions also encompass Kaposi's sarcoma, metastasis, lymphoma, and pyogenic granuloma [13].

ALHE exhibits diverse clinical presentations, resulting in various prebiopsy impressions. In a study of 116 patients by Olsen and Helwig, the most common prebiopsy diagnoses were epidermal cysts and angiomas. The lesion's consistency, color, form, size, growth rate, and other characteristics can result in prebiopsy diagnosis of lymph nodes, pyogenic granulomas, scalp nodules, and lipomas [14].

While serum hypereosinophilia is observed in 21 % of cases, it is not essential for diagnosis. Radiologic studies, such as MRI or angiography, may be necessary to assess the lesion's extent [15]. In our case, the clinical presentation and MRI report led us to a prebiopsy diagnosis of hemangioma.

Microscopically, ALHE is characterized by the proliferation of vascular channels accompanied by a mixed inflammatory infiltrate [1]. This is observed as nests and cords of endothelial cell proliferations with mixed lymphocytes, plasma cells and eosinophils, accompanied by hemorrhage and proliferation of thick and thin-walled blood vessels. Endothelial cells have a hobnail-like appearance with large vesicular nuclei and acidophilic, sometimes vacuolated cytoplasm. Despite visible mitoses, anomalies or anaplasia are not present [16].

Given ALHE's benign course, observation is reasonable if the patient is asymptomatic. However, treatment remains challenging due to the lack of substantial evidence supporting various proposed therapies, such as electrodessication, surgical excision, Mohs surgery, oral propranolol, cryotherapy, topical or systemic corticosteroids, topical tacrolimus, imiquimod or laser therapy [1,3,17].

Because ALHE is a rare disease, treatment recommendations are primarily based on case reports and retrospective investigations. Surgical excision is the standard therapeutic approach and has the lowest failure rate (40.8 %) as defined by recurrence or incomplete resolution.

Though generally benign, ALHE has been linked to lymphoproliferative disorders, with rare cases showing monoclonal T-cell processes and associations with peripheral T-cell lymphoma [6]. The likelihood of recurrence is highest when ALHE is linked to many lesions, bilateral lesions, a longer disease duration, symptomatic lesions, and an earlier age of onset [2]. In our case, surgery was performed, and during a two-year follow-up, there were no signs of recurrence.

## 4 Conclusion

This case report highlights the diagnostic challenges and complexities associated with angiolymphoid hyperplasia with eosinophilia (ALHE), a rare benign vascular tumor that can easily be misdiagnosed as other vascular lesions. Despite initial MRI findings suggesting hemangioma, the definitive diagnosis was made through histopathological examination. Surgical excision remains the treatment of choice, with a low recurrence rate when properly managed. Our patient's successful outcome over a two-year follow-up period underscores the importance of accurate histopathological evaluation in guiding appropriate treatment and improving patient prognosis in cases of ALHE.

## CRediT authorship contribution statement

*Tahmineh Tahouri:* Led the drafting and writing of the manuscript, collected and analyzed the patient data, and played a key role in the conception and design of the case report.

*Ehsanollah Rahimi-Movaghar*: Coordinated the research process, conducted the literature review, and provided critical revisions to the manuscript. Managed all communications with the journal as the corresponding author.

*Sahand Hedayati-Omami:* Contributed to the data analysis and interpretation, supported the writing process by reviewing the manuscript for intellectual and scientific content, and provided additional insights into the clinical management of the case.

*Maryam Moeini:* Assisted with data collection, contributed to the analysis and clinical interpretation, and helped refine the discussion section by incorporating recent research findings into the case report.

All authors reviewed and approved the final version of the manuscript for submission and agree to be accountable for all aspects of the work.

## Consent

Written informed consent was obtained from the patient for publication of this case report and accompanying images. A copy of the written consent is available for review by the Editor-in-Chief of this journal on request.

## Ethical approval

Case reports are exempt from ethical approval in our institution.

## Guarantor

Ehsanollah Rahimi Movaghar. M.D

## Provenance and peer review

Not commissioned, externally peer-reviewed.

## Funding

This study did not receive any specific grant or funding.

## Registration of research studies

Not applicable.

## Declaration of competing interest

The authors declare that they have no conflict of interests.
